# Load effect of visual working memory on distractor interference: An investigation with two replication experiments

**DOI:** 10.3758/s13421-024-01610-y

**Published:** 2024-07-22

**Authors:** Hasan Gunduz, Arzu Ozkan Ceylan

**Affiliations:** 1 Department of Psychology, Adana Alparslan Türkeş Science and Technology University, Adana, Türkiye; 2 Department of Psychology, Hacettepe University, Ankara, Türkiye

**Keywords:** Visual short-term memory, Working memory, Selective attention, Load theory, Replication

## Abstract

Konstantinou et al. (Experiment 1B; *Attention, Perception, & Psychophysics*, *76*, 1985–1997, 2014) reported that an increase in visual short-term memory (VSTM) load reduced distractor interference in the flanker task. Yao et al. (Experiment 3; *Attention, Perception, & Psychophysics*, *82*, 3291–3313, 2020) replicated the design of Konstantinou et al.’s experiment and showed that the VSTM load did not modulate the distractor interference effect, contradicting the original findings. However, it is unknown whether differences in task-design between the two experiments contributed to the inconsistent results. Therefore, we first replicated the original two studies with Experiment 1 (N = 54) and Experiment 2 (N = 54) and performed a statistical comparison between the data from these two experiments. In a third experiment (N = 28), we incorporated articulatory suppression into the design to exclude possible effects of verbalization. According to the ANOVA analyses, the VSTM load did not change the level of distractor interference in all three experiments, indicating that differences in task design alone do not explain the inconsistency.

**Supplementary Information:**

The online version contains supplementary material available at 10.3758/s13421-024-01610-y.

## Introduction

“How does selective attention ignore the irrelevant stimuli?” has been one of the key questions in the attention literature for many years (see Driver, 2001, for a detailed review). Lavie (1995) proposed that the perceptual load of the target task is an important variable that determines to what extent a distractor can interfere with the target behavior. According to the Load Theory of Attention (Lavie, 1995), perception has limited capacity, and this capacity is used automatically for all stimuli in the environment, with target-relevant ones taking precedence. Therefore, if a task involves a high perceptual load, the target-relevant stimuli engage the whole perceptual capacity, and no capacity is left for the irrelevant ones. If the perceptual load of the task is low, on the other hand, the remaining capacity spills over automatically to process the irrelevant stimuli such as distractors. In the former case, the selection between a target and a distractor is made perceptually at an early stage of information processing, and the distractor for which no perceptual resources are allocated has no or little effect on task performance. However, in the latter case, the distractor cannot be perceptually excluded, and attentional selection requires active cognitive control (De Fockert et al., 2001; Lavie et al., 2004; Lavie, 2010). Such an effect pattern of perceptual load on selective attention has been consistently demonstrated in a number of studies (e.g., Forster & Lavie, 2007; Lavie, 1995; Lavie & Fox, 2000; Murphy & Greene, 2015; Schwartz et al., 2005; Wang et al., 2016).

The active cognitive control functions are needed to prevent perceived distractors from taking control of the behavior in low perceptual load situations. Working memory (WM) is one of these higher-level cognitive control functions and can be described as a system of components holding and processing a limited amount of information temporarily for use in support of cognition and action (Adams et al., 2018; Baddeley et al., 2021). Different WM models have been proposed such as the Embedded-Processes Approach (Cowan, 1999; Cowan et al., 2021), Time-Based Resource Sharing Model (see Barrouillet & Camos, 2021) and Multicomponent Model (see Baddeley, 2012). These models differ from each other in terms of degree of modularity, how important they consider attention in storing information, and whether they are idiographic or nomothetic (Adams et al., 2018). An important reason for proposing various WM models with different characteristics is its interaction with many different cognitive processes such as cognitive control, language acquisition, problem solving, creative thinking. WM is also thought to be required for selective attention because it is needed to actively maintain goal-related information and prioritize relevant stimuli over irrelevant ones during task performance. Therefore, if the resources of WM are not available to exert priority-based control, the interference of the distractors on the task should increase due to the poor distinction between the distractor and the target (De Fockert, 2013; De Fockert et al., 2001). Indeed, Lavie et al. (2004) reported that in the condition of maintaining six digits in WM (high WM load condition) compared to one (low WM load condition) while performing a flanker task, the interference of the distractor was greater. In sum, decreasing cognitive control ability by increasing working memory load leads to enhanced processing of low-priority/distracting stimuli (see Brand-D’Abrescia & Lavie, 2008, and Gunduz et al., 2022, for the load effect of other cognitive control functions).

According to the more recent and updated version of the Multicomponent Model of WM, WM is typically characterized as having four basic components, which are defined as the central executive, the phonological loop, the visuospatial sketchpad, and the episodic buffer (Baddeley, 2012). The phonological loop component functions as a repository for stimulus representations to be retained verbally with the help of vocal repetitions in the absence of that stimulus. Similarly, the visuospatial sketchpad is defined as a component that allows visual and spatial information to be stored in WM. These components can receive the support of long-term memory with the help of the episodic buffer component when necessary. The central executive, on the other hand, is a “manager” deciding through which components the representations of stimuli will be maintained, and monitors and manipulates the process along the way (Baddeley, 1992, 2012; Baddeley & Logie, 1999). When the representations of a stimulus are stored in WM verbally with the phonological loop, the storage area of these representations is associated with the anterior regions (e.g., Curtis & D'Esposito, 2003; D'esposito et al., 1995, Yan et al., 2021), and when they are stored visually, they are associated with the posterior regions (e.g., Bettencourt & Xu, 2016; Gayet et al., 2018). Therefore, depending on whether a representation of a stimulus is verbal or visual, the areas related to the resources used by WM on the cortex may differ.

In most of the studies on WM load and selective attention interaction, WM load has been manipulated using verbal stimuli such as numbers or letters (e.g., De Fockert et al., 2001; De Fockert & Bramner, 2011; Lavie et al., 2004; Lavie & De Fockert, 2005). However, the effect of loading visual WM or visual short-term memory (VSTM) on the selectivity of attention may not be similar to the effect of loading verbal WM. Therefore, more recent studies have been focusing on whether the effects of WM load are domain-general by loading the WM with visual stimuli.

In one of the very first studies on VSTM load, Konstantinou et al. (2014) investigated whether the efficiency of distractor rejection changes as a function of the load of VSTM. The load-dependent flanker effect was investigated during both encoding (Experiment 1A, Konstantinou et al., 2014) and maintaining (Experiment 1B, Konstantinou et al., 2014) of the colored squares. During the experiments, participants were first presented with one (low load) or four (high load) colored squares at nine different hypothetical positions in the middle of the screen. Participants were asked to keep these color(s) and their positions in mind. Colors were presented at the same time as (Experiment 1A) or before the flanker task (Experiment 1B). The participants were then presented with a single color and asked whether it was the color they had seen in the position where this single color had been presented. Researchers showed that just like in the case of the perceptual load effect, increasing load during both encoding and maintenance of visual stimuli reduced the compatibility effect on the flanker task. They concluded that load effect of WM on selective attention is not domain general and that common sensory visual representation sources were taxed by visual perception, encoding, and visual maintenance (Konstantinou et al., 2014). There has also been some other evidence supporting the notion that the VSTM load effect on the selective attention task is similar to perceptual load effects (e.g., Konstantinou & Lavie, 2013; Roper & Vecera, 2014).

However, studies manipulating VSTM load have not yielded consistent results. Yao et al. (2020), for example, published a study in which they conducted nine experiments and concluded that VTSM load did not influence the effect of distractor interference. Among these nine experiments, it is worth noting that the third experiment, which aimed to replicate Experiment 1B of Konstantinou et al. (2014), failed to reproduce the critical findings of the original study. In their attempt to explain the inconsistent results, Yao et al. (2020) cited some studies indicating that the visual information coming to the sensory areas can be stored not in the visual but in other areas of the cortex after being encoded (e.g., Xu, 2017). However, it is necessary to highlight that there were some differences between the experimental procedure of Experiment 3 of Yao et al. (2020) and the original study (Experiment 1B of Konstantinou et al., 2014). For example, compared to the original study, in Experiment 3 of Yao et al. (2020), the stimulus sizes in the memory set were larger, the duration of the fixation display after the presentation of the memory set display and the duration of the flanker display were both longer, while the duration of the flanker response display was shorter, and the trials did not begin with the participant response. Also, there was no auditory feedback for incorrect flanker response in Experiment 3 of Yao et al. (2020) contrary to the original study. It is not known whether these variables are responsible for the inconsistent results. However, there is a body of research indicating that they might have affected the results.

For instance, Lee and Jeong (2020) conducted a study to explore the impact of the width of the attentional zoom area of the memory set display on the distractor interference. They hypothesized that an increase in the size of the zoom area will decrease the proportion of attentional resources distributed within the field, which will in turn increase the effect of distractors (see Eriksen & St. James, 1986). In a classical visual working memory load task, an increase in load often results in an increase in the visual attentional field, whether it is intended or not, simply because more stimuli are presented. So, in such classical experiments, it is difficult to determine the exact source of the effect since both WM load and the attentional zoom area increases at the same time. Lee and Jeong (2020), through a series of experiments, dissociated the effects of these two factors by manipulating both the VSTM load and the width of the attentional zoom area separately. They revealed that VSTM load alone did not have a significant effect on the interference caused by distractors, but the width of the attentional zoom area did.

The researchers emphasized the importance of the width of the attentional zoom area especially in the high load condition, and cited previous studies showing that the distractor effect was not modulated by VSTM load if there was a relatively narrow zoom area in the high load condition. It was also reported that this effect could be observed in memory task designs that required a large attentional zoom area in the high load condition. This indicates that increasing load may increase the distractor effect, when a memory task demands a large attentional focus area, especially in higher load conditions. This may be important in explaining the inconsistency between the two studies (Konstantinou et al., 2014, and Yao et al., 2020). In both studies, there were colored squares presented in nine possible positions (3 × 3 matrix) in the middle of the screen at both low and high load conditions. However, in Yao et al.'s experiment, the increase in the size of the colored squares also led to an increase in the size of the 3 × 3 matrix, therefore increasing the width of the attention area to be focused on, especially under high load conditions. Considering these, “the perceptual load-like” effect found by manipulating WM load within a narrow area in Konstantinou et al.’s study may not have been present in Yao et al.’s study.

The difference in stimuli sizes also seems to be critical when we consider the Mexican hat model (Müller et al., 2005) according to which the spatial distribution of the attentional field is not linear. In other words, attentional resources do not decrease linearly while moving away from the center of the focused area. Müller et al. (2005) have revealed there are different areas of “suppression” and “attention” surrounding the focus of attention. In their trials, attention seems to decrease while moving away from the center of the focused area at first, as expected; however, after it functionally disappears (suppression area, where the stimuli seem to be unnoticed), it makes a come-back after some point – although weaker – (another attention area, where the stimuli are attended again), then finally it starts to disappear again (final suppression area), resembling a Mexican hat’s curve in an attention graph, thus the model’s name.

Ahmed & De Fockert (2012) showed that the width and location of these “suppression” and “attention” areas may vary according to the width and location of the focus of attention, and working memory capacity. Any difference in the width of the area where attention needs to be focused will change both the location and the width of the “attention” and “suppression” areas surrounding this focus of attention. The original experiments (Konstantinou et al., 2014, and Yao et al., 2020) differ from each other in the distance they had between the focus of attention, which is determined by low and high load condition, and the distractor because of the difference in the size of the colored squares. This may have caused both the distractor and the target to correspond to different areas of “suppression” and “attention” in these two experiments. Thus, in one task the distractor may have been in a location that corresponds to a strong attentional area, while in another it may have corresponded to a strong suppression area, which may have changed the load pattern of VSTM. Therefore, implications of the Mexican hat model, along with the aforementioned issue regarding the width of the attentional zoom area, make the size of the presented colored squares possibly an important factor for explaining inconsistent results.

Another factor potentially explaining the inconsistency may have to do with the difference in display durations. Roper and Vecera (2013) proposed that variations in the presentation time of the flanker task might alter the encoding demand, which in turn could impact the perceptual load of the task. The extended display duration in Experiment 3 of Yao et al. (2020) might have affected the potential interaction between perceptual load and VSTM load. The fact that the participant had more time for the flanker display may have reduced the perceptual demand of the task by providing more exposure to the task. Moreover, having both bigger squares in memory set and a longer retention interval may have made Yao et al.'s working memory task perceptually less demanding compared to Konstantinou et al.'s task. Indeed, Yao et al. (2020) noted the reason for having bigger squares was to make the encoding process less demanding for the participants. Furthermore, it was shown that prolonging the retention interval helped the encoding process to be completely completed during this time, and the residual perceptual effect of encoding was eliminated for the subsequent task (Guo et al., 2021). The longer retention interval and the bigger squares may have resulted in faster removal of possible perceptual effects, especially in the high load condition, and this was not reflected in the attention task.

Therefore, the primary objective of the study was to investigate whether the divergent outcomes between Experiments 1B of Konstantinou et al. (2014) and Experiment 3 of Yao et al. (2020) could be explained by differences in their task designs. Since there were multiple differences between the designs of the experiments, and the effects of these differences may differ when manipulated individually versus in combination, exact replications of both studies (see *Method* section for the slight differences between our replication experiments and the originals) were conducted. If the dissimilarities between the task designs were indeed explanatory factors for the inconsistent results, one would anticipate finding similar outcomes to the original studies as a result of the replication attempts. In other words, it was expected that a higher VSTM load would diminish the interference effect in Experiment 1 (replication of Experiment 1B of Konstantinou et al., 2014), but have no effect on the interference effect in Experiment 2 (replication of Experiment 3 of Yao et al., 2020). With these two replication experiments, we also investigated the effect of the VSTM load on distractor interference for which the literature seems to fail in providing consistent results so far.

## Experiment 1

In Experiment 1, we aimed to replicate Experiment 1B of Konstantinou et al. (2014).

### Method

#### Participants

The number of required participants was calculated via G-Power (Faul et al., 2007). Accordingly, the number of participants required to detect the interaction effect reported with *ƞ*^2^ = 0.20 effect size in Experiment 1B of Konstantinou et al. (2014), with a type 1 error rate of 5% and a power of 0.95, was determined to be 54. After collecting data from the first 54 individuals, we investigated for each participant whether the VSTM load manipulation created the expected load difference. For this, Cowan's *K* (Cowan et al., 2005; K*(Hit rate – False Alarm rate; K = *Number of stimuli presented in the memory set*)) was calculated for each memory load condition using corresponding hit and false alarm rates. This value is often used to estimate the amount of information each participant can retain in working memory under different load conditions. Before calculating this statistical value, the correction suggested by Macmillan and Kaplan (1985) was applied to the hit and false alarm rates, which were either “0” or “1.” Accordingly, new values were assigned using the *(n-0.5)/n* formulation for the values of “1” and *(0.5/n)* formulation for the values of “0.” The “n” in this formulation corresponds to the total number of trials with the probability of having a hit or false alarm decision. As a result of this calculation, the data of the participants whose Cowan's *K* value for the low VSTM load condition was higher than the value for the high VSTM load condition were excluded from the analysis (n = 5) because they were considered to maintain less representation in the high working memory load condition than in the low load condition.

In the investigation conducted with the remaining participants, trials with the reaction time (RT) of the flanker task outside the range of 100–1,500 ms were excluded from the data of the participants (see Forster & Lavie, 2007). Then, the trials of RTs outside the ± 3 z-score range were also excluded from the participant's data. This procedure was followed for each of the four different condition combinations (i.e., high load/compatible, low load/compatible, high load/incompatible, and low load/incompatible). The number of trials in which each participant responded correctly to both the flanker and the memory task in each condition was examined. If this number of trials was less than half of the total number of trials for any condition, the data was not included in the analyses. In other words, it was determined as a prerequisite for participants to have at least 48 trials for flanker RT analysis in each condition combination. Therefore, five more participants with less than 48 correct attempts were eliminated. Thus, the data of ten participants in total were removed from the data set. In order to reach the predetermined 54, new data were collected from ten more participants, all of whom satisfied the inclusion criteria.

None of the participants reported any color discrimination problems, vision problems, or any psychological/neurological disorders. As a result, analyses were conducted with data from 54 individuals (29 male and 25 female, *M*_*age*_ = 21.24 years, *SD* = 1.86 years, Min/Max = 18/30, Median = 21).^1^

We find it useful to make the following explanations about the elimination criteria. Although Cowan's *K* value was calculated in the original study, it is understood that no elimination was made according to this value even though there was at least one participant with a higher Cowan’s *K* value in the low load condition compared to the high. In addition to this, it is not known whether an elimination criterion based on the number of trials was used in the original study. In Experiment 3 of Yao et al. (2020), on the other hand, Cowan’s *K* was not calculated at all. However, we think that it is necessary to make an elimination based on the Cowan's *K* value, since there is a possibility that the result pattern may be reversed if a participant’s WM capacity is loaded more in the low load condition. Moreover, we believe that representing each condition with as many trials as possible will increase the reliability of the results. In addition to this, since our main aim in the study was to investigate the possible effect of design differences in the tasks, we have tried to increase the attributability of the results to these differences by treating the data in the same way in both our replication experiments.

#### Tasks/paradigms

The experiment was designed using E-Prime 2.0 (E–Prime 2.0 Professional, Pittsburg, PA, USA) and run on an Asus PC (15.6 in., 1,920 × 1,080 resolution, 16 GB RAM ve i7-6700HQ processor 2.60 GHz). The whole experimental procedure was the same as in Experiment 1B of Konstantinou et al. (2014), and detailed below.

##### Flanker task

In the flanker task based on the response competition paradigm (Eriksen & Eriksen, 1974), two different target letters (“X” or “Z” subtending 0.6° × 0.4°) were presented on a hypothetical circle (2° in radius) of small black dots. The probability of the target letter being in six different positions on the circle was equal. Outside of this circle, a distracting letter (subtending 1° × 0.6°) was presented 3.5° left or right from the center of the circle. The probability of the distractor letter being compatible (the distractor “X” when the target was “X”) or incompatible (the distractor “Z” when the target was “X”) with the target letter was equal. The flanker display, where the target and distractor were presented, remained on the screen for 150 ms. This display was followed by another display with “?” at the center. Throughout this 1,850-ms lasting “?” display, participants were asked to respond as quickly as possible to the target letter by pressing “1” for “X” and “2” for “Z”.^2^ These response keys located on the top left of the keyboard were also labeled as “X” and “Z.” An auditory “beep” tone was used as feedback for incorrect flanker responses.

##### Memory task

**Fig. 1:**
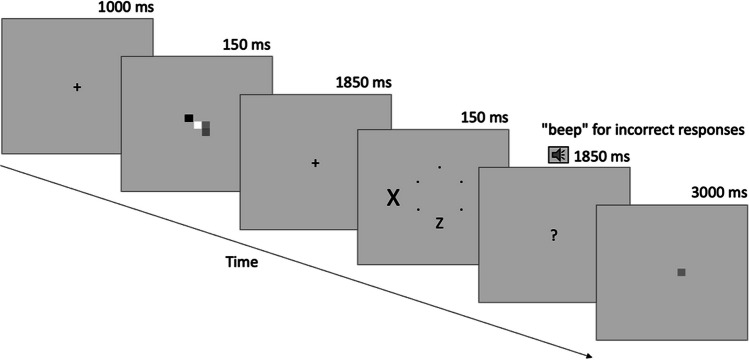
Task procedure of Experiment 1. Each trial began with participant’s response. Figures are not drawn to scale

Each trial began with a fixation cross " + " display lasting 1,000 ms. The participants were asked to start each trial by pressing the "spacebar" key when ready.^3^ Following this, a memory set display consisting of one (low load) or four (high load) colored squares (0.38° × 0.38°) randomly placed on a hypothetical 3 × 3 grid (1.38° × 1.38°) in the center of the screen was presented with a duration of 150 ms. All the colors on the screen were different from each other and chosen randomly from black, blue, cyan, green, magenta, pink, red, white, and yellow (see Konstantinou et al., 2014, for original color space and codes).^4^ The background color was determined as mid-gray. Participants were asked to maintain the square(s) in the memory set throughout the retention interval and to decide whether the color of the memory probe square appearing at the end of the interval was the same as or different from the color of the square at the same location in the memory set. The memory set display presented for 150 ms was followed by another fixation display lasting 1,850 ms. After this display, the flanker task summarized above was presented. Therefore, a participant had to maintain the representation of the colored squares for a total of 3,850 ms. The last display lasting for 3,000 ms was the memory probe display, where only one color was presented in one of the positions occupied on the memory set screen. Participants were asked to press "8" to indicate that the square displayed on the probe screen was the “same” color as the square presented in the same location in the memory set before, and "9" to indicate that it was “different.” These response keys, which were on the top right of the keyboard, were labeled as “Same” and “Different.” The correct response for the memory task was “same” in a random half of the trials and “different” in the other half. It was emphasized that the participants did not need to be fast in their response to the memory probe, and the important thing was accuracy. No feedback was given for this memory response. Memory load conditions were presented as eight blocks in the ABBABAAB order for half of the participants, and this order was reversed for the other half of the participants. After completion of two practice blocks of 16 trials each, the participants completed a total of eight blocks, each consisting of 48 trials during the main experiment (see Fig. 1 for task procedure).

## Experimental procedure

The experimental procedure was approved by the ethics committee (Date: 27 October 2020; Number: 35853172–300) and was carried out in accordance with the Declaration of Helsinki. The experimental procedure began with participants providing informed consent. The individuals confirming participation were first asked to complete the questions on the demographic information form. The distance from the computer screen was set as 60 cm and the participants were instructed to maintain this distance as much as possible during the whole experiment. Before starting the computer-based task, the participants were informed about what was expected of them during the task. This information was also summarized on the computer screen at the beginning of the task. After the participants’ questions were answered, they practiced the task. Participants who wanted to practice the task again were given the opportunity to do so. When participants completed the exercise part, they started the main experiment, which consisted of a total of 384 trials. During the experiment, there were a total of three rest intervals, once in every 96 trials. Each of these rest periods lasted a maximum of 2 min or ended with the participant's response indicating their intention to skip the remainder of that resting period. During these rest periods, it was emphasized to the participants that they should keep their distance from the computer as much as possible. After the completion of the experiment, the participants’ questions, if any, were answered and the experimental procedure ended.

### Results

#### Effectiveness of the VSTM load manipulation

All analyses were carried out using the JASP (2021; version 0.16). The mean Cowan's *K* values in the low and high VSTM load conditions were compared with the *t*-test for paired groups. Results indicated that the participants' mean Cowan's *K* value for the low VSTM load condition (*M* = 0.923, *SD* = 0.055) was significantly lower than the Cowan’s* K* value for the high VSTM load condition (*M* = 2.189, *SD* = 0.633) as expected (*t*(53) = -15.309, *p* < 0.001, *d* = 2.817).

Error percentages for each participant's memory decision were calculated. We tested whether the mean error rate in the low VSTM load condition (*M* = 3.993, *SD* = 2.946) differed significantly from the rate in the high VSTM load condition (*M* = 23.373, *SD* = 8.544) through the *t*-test for paired groups. According to the results, the error rates differed as a function of the VSTM load (*t*(53) = -19.015, *p* < 0.001, *d* = 3.032). These two analyses revealed that the VSTM capacity was occupied as expected.

#### Flanker task performance

##### Error rates

Error percentages in the flanker task of the trials in which the participants made the correct memory decision (Table 1) were analyzed with a 2 (VSTM Load: Low and High) × 2 (Distractor Compatibility: Compatible and Incompatible) ANOVA for repeated-measures.

**Table 1:** Means of reaction times and error rates in the flanker task (Experiment 1)

Conditions	Distractor compatibility								
	Compatible			Incompatible			Interference		
	RT(ms)	Err(%)	Err(Arc)	RT(ms)	Err(%)	Err(Arc)	RT(ms)	Err(%)	Err(Arc)
Low VSTM load	658.162 (124.487)	1.283 (1.672)	0.084 (0.078)	728.399 (123.475)	4.378 (4.310)	0.183 (0.110)	70.237 (40.065)	3.095 (3.556)	0.099 (0.100)
High VSTM load	660.398 (126.111)	1.530 (1.832)	0.092 (0.084)	734.450 (133.107)	4.924 (3.941)	0.202 (0.100)	74.053 (49.523)	3.394 (3.854)	0.110 (0.111)

RT = reaction time, Err = error, Arc = arcsine transformation, ms = millisecond. Standard deviations of the means are given in parenthesis

Arcsine transformation was applied to the error rates due to non-normal distribution (Duran, 1997) through “acin(square rootX)” formulation in which “X” corresponded to the original error rates ranging from 0(0%) to 1(100%). Interpretations of the results were made given the fact that the increase in the converted values ​​corresponded to the increased error percentage.

Analysis revealed that the main effect of the VSTM load was not significant (*F*(1, 53) = 1.541, *p* = 0.220, *ƞ2p* = 0.028). The main effect of distractor compatibility, on the other hand, was significant (*F*(1, 53) = 69.464, *p* < 0.001, *ƞ2p* = 0.567). Accordingly, the mean error rate in incompatible trials was higher than the error rates in compatible trials (*MD* = 0.104, *SE* = 0.013). The interaction effect was not significant (*F*(1, 53) = 0.591, *p* = 0.446, *ƞ2p* = 0.011).

##### Reaction times

The mean RTs in the flanker task of the trials, in which both the memory decision and the responses to the flanker task were correct (Table 1), were analyzed with 2 (VSTM Load: Low and High) × 2 (Distractor Compatibility: Compatible and Incompatible) ANOVA for repeated measures.

The main effect of the VSTM load was not significant (*F*(1, 53) = 0.435, *p* = 0.512, *ƞ2p* = 0.008), indicating that the increased memory load did not change the participants' average RTs. On the other hand, the main effect of distractor compatibility was significant (*F*(1, 53) = 170.358, *p* < 0.001, *ƞ2p* = 0.763), indicating that reactions to incompatible trials were slower than the reactions to the compatible trials (*MD* = 72.145, *SE* = 5.527). More importantly, the interaction effect was not significant (*F*(1, 53) = 0.519, *p* = 0.475, *ƞ2p* = 0.010).

**Fig. 2:**
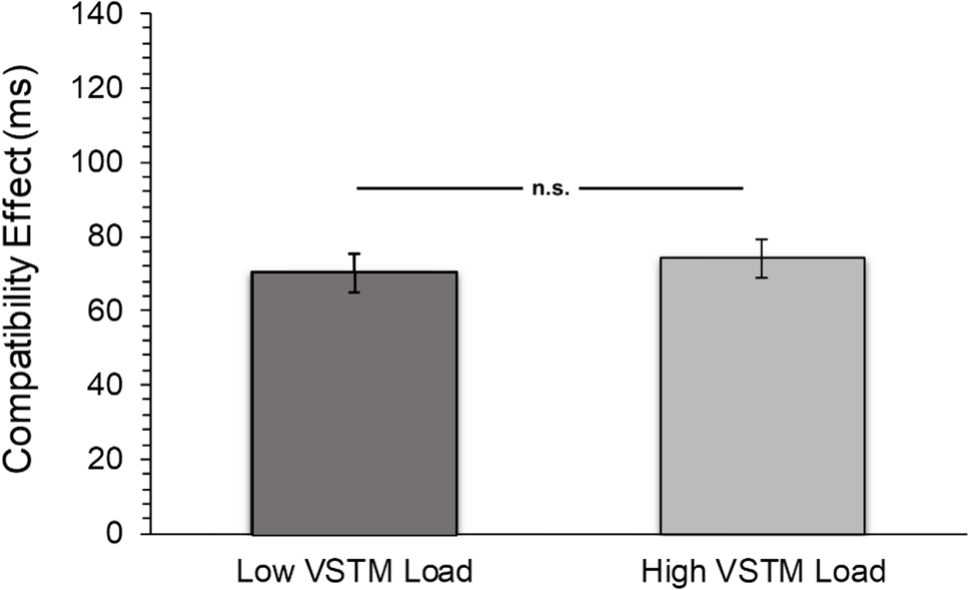
Compatibility effect in flanker task under different VSTM load conditions (Experiment 1). Error bars show standard errors. n.s. = non-significant

The effect of VSTM load on the distractor interference (RT) was also investigated through Bayesian analysis (Fig. 2). The prior distribution parameter of the effect size for the Bayesian *t*-test for dependent groups was determined using the default option (Cauchy, *r* = 0.707) in JASP. H1 alternative hypothesis was determined as two-tailed: “The interference effect on RT is different between the two VSTM load conditions.” The estimated Bayes factor value (BF01 = 5.27, %H = 0.011, *p* = 0.475, %95 GA = [-0.352, 0.166]) for the difference in the compatibility effect between the two VSTM load conditions revealed that the probability of the data supporting the null hypothesis model was 5.27 times higher than the probability of supporting the H1 alternative hypothesis model. Our data provided moderate evidence (Jarosz & Wiley, 2014) for the null hypothesis.

## Experiment 2

Yao et al. (2020) aimed to replicate Experiment 1B of Konstantinou et al. (2014) in their third experiment. However, there were some differences between the experimental procedures of these two experiments. In Experiment 3 of Yao et al. (2020): (i) The colored squares were larger; (ii) the duration of flanker display was 50 ms longer; (iii) the duration of flanker task response screen was 50 ms shorter; (iv) no feedback was provided for incorrect responses in the flanker task; (v) the fixation display following memory set was presented 50 ms longer; (vi) the number of blocks for the working memory load condition was half the number of the original study; (vii) the identity of one of the target letters for the flanker task was different (using “N” instead of “Z”); (vii) unlike the original study, each trial started automatically, not with a participant response; (ix) the duration of the memory probe display depended on the participant’s response, up to 3,000 ms; however, in the original study, the probe display was alive for 3,000 ms even if the participant responded; (x) different keys were used to respond to the flanker task; (xi) the color pink was not used in order to improve color distinction. We retained these differences in our Experiment 2, which aimed to replicate Experiment 3 of Yao et al. (2020). However, we kept the response keys the same as the response keys in our Experiment 1.

### Method

#### Participants

As in Experiment 1, 54 participants were planned to be recruited for this experiment. The elimination criteria in Experiment 1 were also used for this experiment. Accordingly, six people were eliminated after examination of Cowan's *K* values. In addition, the data of two participants with less than 24 (50%) analyzable trials for any condition combination and of one participant whose responses were not recorded for technical reasons were not included in the analysis. In order to reach the number 54, data were collected from nine more participants. None of the participants reported any color discrimination problems, vision problems, or any psychological/neurological disorders. Analyses were performed with data from 54 participants (26 male and 28 female, *M*_age_ = 21.31 years, *SD* = 1.78 years, Min/Max = 18/30, Median = 21).

#### Tasks/paradigms

##### Flanker task

The characteristics of the flanker task were the same as in Experiment 1. However, in Experiment 2, the presentation time of the flanker display was 50 ms longer, and the presentation time of the response display was 50 ms shorter as mentioned earlier. The target letters were labeled as "X" and "N." Here, the participants responded by pressing “1” for “X” and “2” for “N.”

##### Memory task

**Fig. 3:**
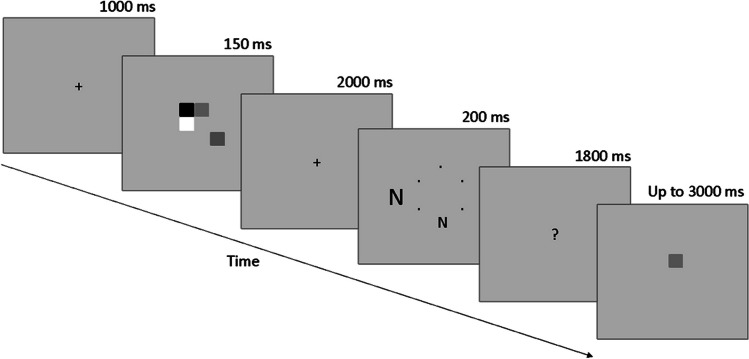
Task procedure of Experiment 2. Each trial began automatically. Figures are not drawn to scale

Each trial began with a fixation cross " + " display lasting 1,000 ms. Following this, a memory set display was presented for a duration of 150 ms. The dimensions of the memory set stimuli presented in the center of the screen were different from the dimensions of the original study (Experiment 1B of Konstantinou et al., 2014). The memory set display consisted of one (low load) or four (high load) colored squares (0.8° × 0.8°) randomly placed on a hypothetical 3 × 3 grid (2.8° × 2.8°). The duration of the fixation display following the memory set display was 2,000 ms. The total duration of the memory retention interval was 4,000 ms. Memory load conditions were presented as blocks in ABBA order, and these conditions were counterbalanced between participants. After completion of two practice blocks of 16 trials each, the participants completed a total of four blocks of 48 trials each during the experiment. All the instructions and the response keys were the same as in Experiment 1 (Fig. 3).

#### Experimental procedure

All the experimental procedures were very similar to the procedure of Experiment 1. However, during Experiment 2, after 96 trials, only one rest display lasting 2 min was presented. After the completion of the experiment, the participant's questions, if any, were answered and the experimental procedure ended.

### Results

#### Effectiveness of the VSTM load manipulation

The mean Cowan's *K* values corresponding to the low and high VSTM load conditions were compared with the *t*-test for paired groups. According to the results, the mean Cowan's *K* value for the low VSTM load condition (*M* = 0.905, *SD* = 0.073) was lower than the mean Cowan’s *K* value for the high VSTM load condition (*M* = 2.113, *SD* = 0.803). This difference was statistically significant (*t*(53) = -11.530, *p* < 0.001, *d* = 2.118).

In addition, the mean error rates for two memory load conditions (low load: *M* = 4.880, *SD* = 4.096; high load: *M* = 24.055, *SD* = 10.354) were compared with the *t*-test for paired groups and it was found that these rates differed significantly from each other (*t*(53) = -15.635, *p* < 0.001, *d* = 2.435). All the analyses reported above indicated that memory load manipulation was effective for Experiment 2.

#### Flanker task performance

##### Error rates

The error percentages in the flanker task of the trials in which the participants made the correct memory decision (Table 2) were analyzed with 2 (VSTM Load: Low and High) × 2 (Distractor Compatibility: Compatible and Incompatible) ANOVA for repeated measures. Arcsine transformation was applied for the error percentages due to non-normal distribution.

According to the results, the main effect of the VSTM load was significant (*F*(1, 53) = 9.697, *p* = 0.003, *ƞ2p* = 0.155) indicating that increasing memory load increased the average error percentages of the participants in the flanker task (*MD* = 0.039, *SE* = 0.013). The main effect of distractor compatibility was also significant (*F*(1, 53) = 24.239, *p* = 0.000, *ƞ2p* = 0.314). Accordingly, the error rate in the incompatible trials was found to be greater than the error rate in the compatible trials (*MD* = 0.083, *SE* = 0.017). Finally, the interaction effect was not significant (*F*(1, 53) = 2.100, *p* = 0.153, *ƞ2p* = 0.038).

##### Reaction times

Mean RTs in the flanker task of the trials, in which both the memory decision and the responses in the flanker task were correct (Table 2), were analyzed with 2 (VSTM Load: Low and High) × 2 (Distractor Compatibility: Compatible and Incompatible) ANOVA for repeated measures.

**Table 2:** Means of reaction times and error rates in the flanker task (Experiment 2)

Conditions	Distractor compatibility															
	Compatible					Incompatible						Interference				
	RT (ms)	Err (%)		Err (Arc)		RT (ms)	Err (%)		Err (Arc)		RT (ms)		Err (%)		Err (Arc)	
Low VSTM load	654.647 (151.626)		2.495 (3.080)		0.117 (0.110)	715.649 (167.045)		4.741 (4.752)		0.183 (0.125)		61.003 (70.796)		2.246 (4.955)		0.066 (0.132)
High VSTM load	658.818 (137.257)		3.018 (2.842)		0.139 (0.108)	736.889 (157.994)		7.460 (5.968)		0.239 (0.146)		78.071 (89.905)		4.443 (5.716)		0.996 (0.166)

RT = reaction time, Err = error, Arc = arcsine transformation, ms = millisecond. Standard deviations of the means are given in parenthesis

According to the results, the main effect of the VSTM load was not significant (*F*(1, 53) = 1.991, *p* = 0.164, *ƞ2p* = 0.036), meaning that the VSTM load did not change the participants' average RTs (*MD* = 12,706, *SE* = 9.004). The main effect of distractor compatibility, on the other hand, was significant (*F*(1, 53) = 52.143, *p* < 0.001, *ƞ2p* = 0.496) indicating that the reactions in the incompatible trials were slower than the reactions in the compatible trials (*MD* = 69.537, *SE* = 9.630). The interaction effect was not significant (*F*(1, 53) = 2.554, *p* = 0.116, *ƞ2p* = 0.046).

**Fig. 4:**
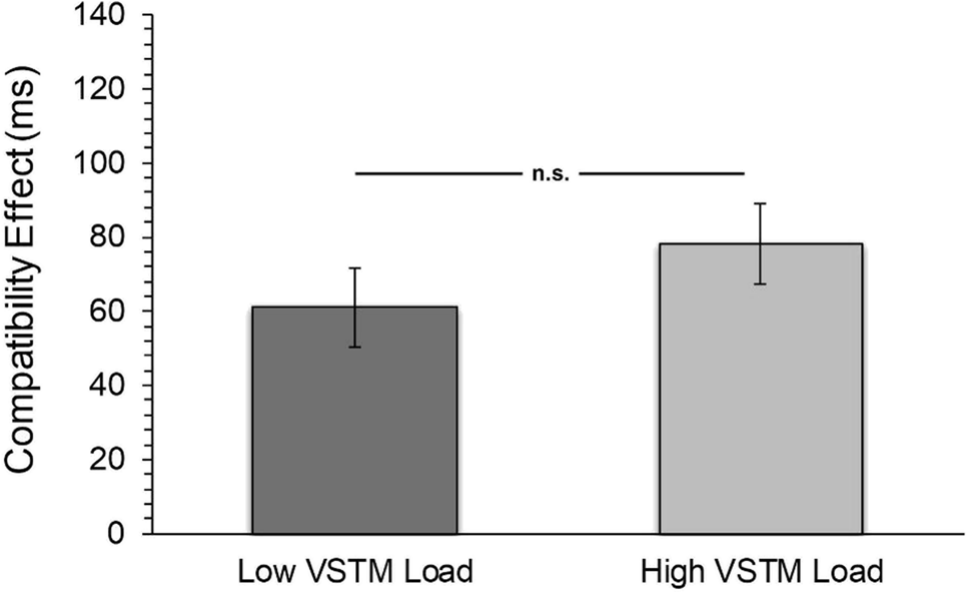
Compatibility effect in flanker task under different VSTM load conditions (Experiment 2) Error bars show standard errors. n.s. = non-significant

Results indicated that VSTM load did not modulate the distractor rejection ability (Fig. 4). A Bayesian *t*-test for paired groups was also conducted as in Experiment 1. The estimated Bayes factor value (BF01 = 2.05, %H = 0.000, *p* = 0.116, %95 GA = [- 0.469, 0.056]) for the difference in the interference effect between the two VSTM load conditions revealed that the probability of the data supporting the null hypothesis model was 2.05 times higher than the probability of the supporting H1 alternative hypothesis model. The data of Experiment 2 provided anecdotal evidence for the null hypothesis model (Jarosz & Wiley, 2014).

#### Comparison of Experiment 1 and Experiment 2

Although the effects obtained in the two experiments had similar patterns, it was yet to be determined whether these results are indeed comparable statistically. Therefore, we combined the data of Experiment 1 and Experiment 2 and created a new independent variable called “Experiment” with two factors: Experiment 1 and Experiment 2. Cowan's *K* values and error rates of the memory task were analyzed by a 2 (Experiment: Experiment 1 and Experiment 2) × (VSTM Load: Low and High) mixed factorial ANOVA. Error rates and RTs of the flanker task were analyzed with a 2 (Experiment: Experiment 1 and Experiment 2) × 2 (VSTM Load: Low and High) × 2 (Distractor Compatibility: Compatible and Incompatible) mixed factorial ANOVA. Since the main interest was to compare the two experiments, only the results corresponding to the main effect of the “Experiment” and the interaction effects including the “Experiment” variable were reported.

#### Memory task performance

Analysis on the error rates of memory task indicated that neither the main effect of Experiment (*F*(1, 106) = 0.485, *p* = 0.488, *ƞ2p* = 0.005) nor the interaction effect were significant (*F*(1, 106) = 0.017, *p* = 0.898, *ƞ2p* = 0.001). Similar results were also found for the Cowan’s *K* values. Accordingly both the main effect of Experiment (*F*(1, 106) = 0.416, *p* = 0.520, *ƞ2p* = 0.004) and the interaction effect were non-significant (*F*(1, 106) = 0.185, *p* = 0.668, *ƞ2p* = 0.002). Results indicated that memory task performance and VSTM capacity occupancy were statistically similar in both experiments.

#### Flanker task performance

Arcsine transformed error rates were analyzed. According to the results, the main effect of the experiment was significant (*F*(1, 106) = 3.950, *p* = 0.049, *ƞ2p* = 0.036), indicating that the error rate in Experiment 2 was higher than the rate in Experiment 1 (*MD* = 0.029, *SE* = 0.015). The interaction effect of VSTM load and Experiment (*F*(1,106) = 2.200, *p* = 0.141, *ƞ2p* = 0.020), and of Distractor Compatibility and Experiment were not significant (*F*(1, 106) = 1.033, *p* = 0.312, *ƞ2p* = 0.010). The three-way interaction was also not significant (*F*(1, 106) = 0.687, *p* = 0.409, *ƞ2p* = 0.006).

Results of the mixed factorial ANOVA on RT revealed a non-significant main effect of Experiment (*F*(1,106) = 0.022, *p* = 0.882, *ƞ2p* = 0.002). The interaction effect of VSTM load and Experiment (*F*(1, 106) = 0.608, *p* = 0.437, *ƞ2p* = 0.006), and of Distractor Compatibility and Experiment were not significant (*F*(1, 106) = 0.055, *p* = 0.815, *ƞ2p* = 0.005). The three-way interaction was also not significant (*F*(1, 106) = 1.235, *p* = 0.269, *ƞ2p* = 0.012).

Although there was a statistically significant effect at the 0.049 level in terms of the flanker error rates, the low effect size (*ƞ2p* = 0.036) indicated that the obtained effect was weak. When the analysis results on both RT and error rates are evaluated together, it can be concluded that there was no difference between the two experiments in terms of flanker task performance .

For the Bayesian analyses on how the compatibility effects at different VSTM load conditions differ between the two conditions of the Experiment variable, the H1 hypothesis was defined as being that there will be a difference between Experiment 1 and Experiment 2 conditions in terms of the compatibility effect calculated for a given VSTM load condition. In this determination, the prior distribution parameter was used as the Cauchy distribution (r = 0.707).

**Fig. 5:**
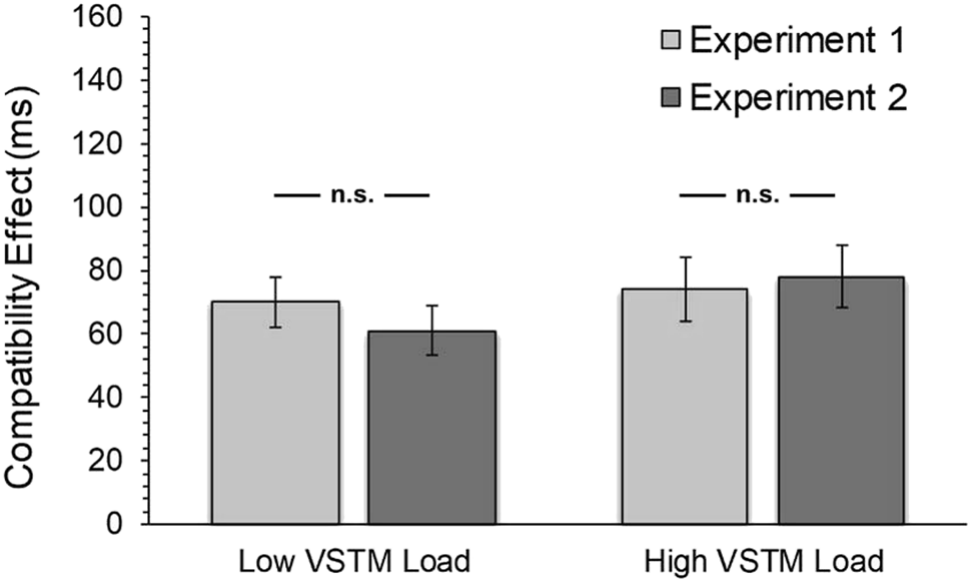
Compatibility effect in flanker task as a function of experiment and VSTM load conditions. Error bars show standard errors. n.s. = non-significant

The Bayesian factor value (BF_01_ = 3.59, %H = 0.022, *p* = 0.406, 95% CI = [-0.213, 0.504]) estimated according to the results of Bayesian t-test analysis for independent groups based on the amount of compatibility effect in the low VSTM load condition revealed that the probability of supporting the null hypothesis of the relevant data was approximately 3.6 times higher than the probability of supporting the alternative hypothesis. The Bayes factor value obtained for the high VSTM load condition (BF_01_ = 4.729, %H = 0.014, *p* = 0.774, 95% CI = [-0.406, 0.306]) revealed that the probability of supporting the null hypothesis of the relevant data was approximately 4.7 times higher than the probability of supporting the alternative hypothesis (Fig. 5).

### Discussion

The main aim of the first two experiments was to examine whether the design-related differences between Experiment 1B of Konstantinou et al. (2014) and Experiment 3 of Yao et al. (2020) might have been the reason for the inconsistent results. Instead of manipulating all the design-related differences one by one, we repeated both experiments separately, preserving all their differences. Moreover, we tested whether the effects found in both our replication experiments differed statistically from each other by combining two datasets.

All the analyses revealed that the levels of distractor interference under each VSTM load condition were comparable and not statistically different from each other. We concluded that design-related differences might not have been responsible for the inconsistency. It is worth noting that in both our replication experiments we took care to collect the data from participants with similar ages and education levels, and applied the same screening/elimination criteria to the data in order to increase the attributability of the results to the design-differences.

This pattern of results was consistent with the results of Yao et al. (2020) but inconsistent with the finding of Konstantinou et al. (2014). Even though it was not a direct aim of our study and there have been some other studies revealing no modulation effect of VSTM load (Lee & Jeong, 2020; Lee & Yi, 2018; Lin & Yeh, 2014; Yao et al., 2020; Zhang & Luck, 2015), we discussed the possible reasons why we failed to replicate the critical interaction effect of Konstantinou et al., (2014; Experiment 1B). Because all these cited experiments had some differences from Experiment 1B of Konstantinou et al. (2014) in terms of experimental task design and their direct aims, the lack of a modulation effect may have been attributed to these differences. However, our Experiment 1 was an almost exact replication in terms of design, yet the critical interaction of RT found in the original study was not obtained. To investigate possible reasons for that, we reviewed the differences between the original study and our replication attempt. These were related to elimination criteria, ages of the participants, and response keys.

In our experiment, contrary to the original experiment, participants were eliminated based on their Cowan’s *K* values and the number of trials they had for RT analysis. Because eliminated participants made too many memory errors, especially in the high load condition, it can be said that the memory capacities of these participants might have also been lower than the others. Therefore, if filling the capacity causes a perceptual load effect, individuals with more limited VSTM capacity may produce a perceptual load-like effect more strongly and their elimination may have caused this effect not to be observed. For this reason, we reanalyzed the data of Experiment 1 with lax elimination criteria. For space considerations, we have provided the results in the Online Supplementary Material (OSM). The results of this analysis with the data of all 64 participants revealed that there was no effect of the strict elimination criteria, as we found a non-significant two-way interaction effect for both error rates and RTs (see *Lax Criteria Analyses of Experiment 1* in the OSM for details of the analysis).

Even if we had no direct evidence, the difference in response keys seems to be a weak possibility for the inconsistency given the fact that the distance between the response keys of the two tasks, thus two hands, in the original experiment was similar to that in Experiment 1.

Although the average ages of the participants in Experiment 3 of Yao et al. (2020) and in our two replication attempts were close to each other, the average age in Experiment 1B of Konstantinou et al. (2014) appears to be slightly higher. The age range of the participants recruited in Experiment 3 of Yao et al. (2020) was 17–27 years, which was similar to the age range in our replication attempts (18–30 years for both replication experiments). In the original study, on the other hand, we were informed that the age distribution of the participants was between 18 and 51 years. Three of these participants were over 30 years old (35, 43, and 51 years of age, respectively). It is unknown what effect these three of the 22 participants in the original study had on the results. Nevertheless, considering the ratio of the number of older participants in the sample was small, and assuming that the necessary actions were taken for the outliers, we thought that the age-related difference should not be the reason for the difference. However, if there is a weakness in this small number of participants in terms of VSTM or perceptual capacity, the impact of age differences on the results would still be open to discussion.

These differences seem to be weak reasons to explain the inconsistency. However, another variable could be the strategic freedom that the participants had in performing the memory task. We questioned whether the task design guaranteed that memory representations were indeed visual. We addressed this issue in the third experiment.

## Experiment 3

Considering the findings of the two replication experiments, and comparison analyses, we inferred that there is no modulation of VSTM load on the efficacy of distractor rejection and the design-related differences seem ineffective in changing the pattern of the results.

The above inference is based on the assumption that load manipulation clearly produces a change in the utilization rate of VSTM resources. However, this assumption may not be entirely reliable due to the issue of verbalization. Maintaining the locations of the colored squares presented in the memory set may have demanded VSTM resources since it is not easy to verbally name the locations. However, colors of the squares can easily be named verbally. In this case, verbal working memory resources associated with frontal areas may have been demanded. Verbalization may have been an adequate strategy for the low load condition, as the stimulus presented in the memory set and the memory probe screen were always in the same position. On the other hand, in the high load condition, where the location of colors became important, it may have been a possibility to use both verbal and visual working memory resources. In addition, the ratio of using visual or verbal working memory resources may have varied from participant to participant. The storage areas correspond to verbal and visual representations, and so the cortical resources they demand are different (e.g., Lee et al., 2013), and there is also some evidence that the load effect for verbal and visual WM may be different (De Fockert, 2013; De Fockert & Bremner, 2011; Konstantinou & Lavie, 2013; Lavie & de Fockert, 2005; Lavie et al., 2004; Lee & Jeong, 2020; Pecchinenda & Heil, 2007; Roper & Vecera, 2014; Yao et al., 2020; Zhang & Luck, 2015).

The strategies used by the participants may have differed both in the original studies and in the experiments we conducted. In this case, referring to the above-mentioned effects as VSTM load effects may not be correct, and we cannot be sure whether design differences can differentiate the effects of visual working memory load. Lee and Jeong (2020) reported that the effect of the width of the attentional window, which we considered an important factor because of design-related differences, manifests itself in tasks in which representations of stimuli are difficult to verbalize.

In the next experiment, we used an articulatory suppression method to prevent maintenance of color representations via verbal codes. By manipulating different experimental designs as two levels of *a within-subjects* variable called “Design,” we also aimed to prevent possible effects of individual differences on the results. The third experiment therefore served several purposes. The first of these was to show the effect of VSTM load on the interference of distractors. The second was to show whether the results pattern would differ from the originals when we were sure that participants visually maintained representations of memory stimuli. The last was to retest whether the design-related differences between the two original experiments led to any difference in results pattern in this kind of experimental design known to demand visual working memory.

We did not formulate specific hypotheses regarding the VSTM load effect on distractor interference. Rather, we preferred to discuss possible explanatory factors on obtained results.

### Method

#### Participants

As in Experiments 1 and Experiment 2, 54 participants were planned to be recruited for the third experiment. Considering that there are eight different presentation orders of the conditions, 56 participants were found. The same elimination criteria were used for this experiment. In the condition of Design 2, it was observed that two participants made 50% or more errors in the memory task, and these participants were excluded from the analysis. The data of the participants whose Cowan's *K* value for the low load condition was higher than the value for the high load condition (n = 1 for Design 1 only, n = 4 for both Design 1 and Design 2, n = 12 for Design 2 only) were excluded from the analyses. Data from nine participants having less than 24 usable trials for flanker RT analyses under any condition (n = 2 for Design 1 only, n = 3 for both Design 1 and Design 2, n = 4 for Design 2 only) were excluded from the analyses. Thus, the number of participants whose data were included in the analyses was 28.

Experiment 3 was notable for its unpredictably high data loss (n = 28, 50%) compared to Experiment 1 (18.52%) and Experiment 2 (16.67%). Instead of acquiring new participants, the suitability of running the analyzes with the number of remaining participants was re-examined. It is known that the results differ according to the method chosen for the sample size calculation in G*Power (Version 3.1; Faul et al., 2007). Since the first two experiments were replication attempts, the "as in Cohen (1988)-recommended" option was used for participant calculation. We re-calculated the required number of participants by changing the parameters in G*Power. The number of participants remaining for the third experiment (*N* = 28) was actually sufficient to find an effect with a power of 0.99 and type 1 error rate of 1% when the *default* option was used in G*Power (Version 3.1; Faul et al., 2007) (see a priori calculation of Yao et al., 2020). Considering both this new calculation and the fact that both Konstaninou et al. (2014, Experiment 1B) and Yao et al., (2020, Experiment 3) included 22 participants in their studies, we decided to conduct the analyses with the data of 28 participants (17 males, 11 females) aged from 18–30 years (*M*_*age*_ = 21.50 years, *SD* = 2.59 years, Median = 21). None of the participants reported any color discrimination problems, vision problems, or any psychological/neurological disorders.

#### Tasks/paradigms

##### Flanker task

As mentioned before, in Experiment 3, the design features of the experiments of both Konstantinou et al., (2014, Experiment 1B) and Yao et al., (2020, Experiment 3) were represented as two levels (Design 1 and Design 2, respectively) of an independent variable called “Design.” Therefore, the characteristics of the flanker task were as in Experiment 1 in half of the experiment and as in Experiment 2 in the other half. However, the target or distractor letters, which were different in Experiment 1 and Experiment 2, were kept constant as "X" and "Z" so that there was no change in responses for the flanker task during Experiment 3.

##### Memory task

In Experiment 3, the characteristics of the memory task were as in Experiment 1 in half of the experiment and as in Experiment 2 in the other half. Although the trials in Yao et al.'s study (2020, Experiment 3) started automatically, all trials started with the participant pressing the "spacebar" in Experiment 3 in order to prevent fatigue. Similarly, while the memory probe display remained for 3,000 ms in the study of Konstantinou et al., (2014, Experiment 1B), regardless of whether the participant responded or not, in the study of Yao et al., (2020, Experiment 3), it was presented for a maximum of 3,000 ms or ended as soon as the participant responded. In our Experiment 3, in order to prevent the duration of the experiment from getting too long, the memory probe display remained up to 3,000 ms or could be skipped as soon as the participant responded.

Blocking was done according to combinations of the design and load conditions. The order of presentation of four different blocks (Design 1/Low, Design 1/High, Design 2/Low, Design 2/High) was made in eight different ways. In all of these presentation sequences, it was ensured that a level of the design variable was not presented until all load conditions of the other level were presented. There were four different presentation orders where the Design 1 condition was presented first (n = 16), and four different presentation orders where the Design 2 condition was presented first (n = 12). In half of these presentation orders, the low VSTM load condition was presented first (n = 15), while the high VSTM load condition was presented first in the other half (n = 13). There were 96 trials in each of the blocks. Participants were presented with a total of 384 trials. The variation in the number of participants in different presentation sequences was due to the fact that Experiment 3 was done with the remaining 28 participants rather than the planned number of the participants (N = 56).

#### Articulatory suppression (AS)

The phonological loop component of WM can temporarily keep verbal information active for vocal repetitions (Baddeley, 2012). In many experiments with the possibility of verbalization, the AS method is used to eliminate the possibility of verbally maintaining the visually presented stimulus (Matsukura & Hollingworth, 2011; Van Lamsweerde & Beck, 2012). The AS usually requires participants to continuously vocalize a word or a number (Murray, 1967; Saito, 1997). It has been reported that the continuous repetition of unrelated information during a verbal memory task disrupts the normal functioning of the phonological loop. It has been stated that such an activity prevents subvocal repetitions of verbal inputs (Baddeley, 2012; Baddeley & Logie, 1999). Alloway et al. (2010) examined the effect of repetition of a single number or sequentially counting numbers on the recall of numbers and reported that both types of vocal suppression methods impair verbal memory performance. More importantly, the visual memory task performance was not affected by AS. These results indicated that encoding and/or maintaining of verbal information is supported by the phonological loop and of visuospatial information is supported by the visuospatial sketchpad components of working memory. This finding is important in terms of showing that when the AS method is used, the affected factor is the support of the phonological loop, which is critical for verbal memory functioning, and that AS does not have a negative effect on the retention of visual information.

In Experiment 3, the AS method was included in the task flow. The WM task started with the participant's response and then the first fixation “ + ” was presented. It was followed by a three-digit number presented for 1,000 ms. In each trial, the number presented was randomly selected among three-digit numbers. Participants were asked to start repeating the number aloud when they saw it and to stop this repetition when they responded for the memory decision (see Allen et al., 2017). For example, if the number displayed on the screen was "753," participants repeated the number aloud as "seven hundred and fifty-three, seven hundred and fifty-three…" starting from the moment they saw the number until the moment they responded to the memory task. Participants were asked not to act too slowly and not to leave any gaps between the repetitions. In the practice phase, how the AS task should be performed was explained and the appropriate tempo was set. To ensure that the AS task was done properly, participants’ verbal responses were monitored throughout the task from a sufficient distance so as not to cause anxiety or distraction in the participant.

#### Experimental procedure

All the experimental procedures were approved by the ethics committee. Participants completed the practice part before starting the task. The number of trials in the practice blocks was 32 (eight for each block). During the experiment, the participants gave their reactions by holding the middle and index fingers of their left hand on the “1” and “2” keys, respectively, and the index and middle fingers of their right hand on the “8” and “9” keys, respectively. Rest displays were presented after each 96 trials. After the completion of the experiment, the participant's questions, if any, were answered and the experimental procedure ended. The duration of the experiment was approximately 75 min.

### Results

#### Effectiveness of the VSTM load manipulation

The mean Cowan's *K* values were analyzed by a 2 (Design: Design 1 and Design 2) × 2 (VSTM Load: Low and High) ANOVA for repeated measures. The main effect of VSTM load was significant (*F*(1, 27) = 105.643, *p* = 0.000, *ƞ2p* = 0.796) and indicated that the mean Cowan's *K* value for the low VSTM load condition was lower than the mean Cowan’s *K* value for the high VSTM load condition (*MD* = -1.027*, SE* = 0.100).

The mean Cowan’s *K* value was also higher for the Design 1 condition compared to Design 2 (*MD* = 0.248*, SE* = 0.042), and this difference was statistically significant (*F*(1, 27) = 34.472, *p* < 0.000, *ƞ2p* = 0.561). Interaction effect was also significant (*F*(1, 27) = 42.580, *p* < 0.001, *ƞ2p* = 0.612). Accordingly, compared to the high VSTM load condition, the Cowan’s *K* value was lower under the low VSTM load condition for both levels of the Design variable (for Design 1: *MD* = 1.286*, SE* = 0.108, *p* < 0.001, *r* = 0.916; for Design 2: *MD* = 0.768*, SE* = 0.108, *p* = 0.000, *r* = 0.807). In addition, a significant difference was found between the Design 1 and Design 2 conditions only under the high VSTM load condition (*MD* = 0.507*, SE* = 0.058, *p* < 0.001, *r* = 0.860), revealing that the Cowan’s *K* value under the Design 1 condition was higher than the value under the Design 2 condition.

Results were similar for error rates. Accordingly, the mean error rates for the low VSTM load condition were significantly lower than the mean error rates for the high VSTM load condition (*MD* = -21.259*, SE* = 1.104), as expected (*F*(1, 27) = 370.507, *p* = 0.000, *ƞ2p* = 0.932). The main effect of the Design was also significant (*F*(1, 27) = 20.907, *p* = 0.000, *ƞ2p* = 0.436), and indicated that the mean error rate in the Design 2 condition was higher than the rate in the Design 1 condition (*MD* = -2.956*, SE* = 0.646). A significant interaction effect (*F*(1, 27) = 48.827, *p* < 0.001, *ƞ2p* = 0.644) indicated that compared to the high VSTM load condition, the mean error rates were lower under the low VSTM load condition in both levels of Design variable (for Design 1: *MD* = -17.634*, SE* = 1.220, *p* = *0.0*00, *r* = 0.941; for Design 2: *MD* = *-*24.884*, SE* = 1.220, *p* = 0.000, *r* = 0.969). It was also revealed by the interaction that only under the high VSTM load condition was the mean error rate significantly lower under the Design 1 condition (*MD* = -6.581*, SE* = 0.829, *p* < 0.001, *r* = 0.837).

All the analyses reported above indicated that memory load manipulation was effective for Experiment 3. This was also valid for both the Design 1 and Design 2 conditions. However, results also revealed that only under the high VSTM load condition was there a difference between the Design 1 and Design 2 conditions in terms of capacity loading.

#### Flanker task performance

##### Error rates

The error percentages in the flanker task (Table 3) were analyzed with a 2 (Design: Design 1 and Design 2) × 2 (VSTM Load: Low and High) × 2 (Distractor Compatibility: Compatible and Incompatible) ANOVA for repeated measures. Arcsine transformation was applied for the error percentages due to non-normal distribution.

According to the results, the main effect of the Design was not significant (*F*(1, 27) = 1.782, *p* = 0.193, *ƞ2p* = 0.062). The main effect of the VSTM load was also not significant (*F*(1, 27 = 2.060, *p* = 0.163, *ƞ2p* = 0.071). The main effect of the Distractor Compatibility, on the other hand, was significant (*F*(1, 27) = 63.129, *p* = 0.000, *ƞ2p* = 0.700), indicating that compared to the compatible condition, the error rates were higher under the incompatible condition (*MD* = *-*0.133*, SE* = 0.017).

Interaction effects were not statistically significant (Design × VSTM load: *F*(1, 27) = 0.542, *p* = 0.468, *ƞ2p* = 0.020; Design × Distractor Compatibility: *F*(1, 27) = 0.023, *p* = 0.881, *ƞ2p* = 0.001, VSTM load × Distractor Compatibility: *F*(1, 27) = 0.003, *p* = 0.958, *ƞ2p* = 0.000; Design × VSTM load × Distractor Compatibility: *F*(1, 27) = 0.518, *p* = 0.478, *ƞ2p* = 0.019).

#### Reaction times

Participants' mean RTs in the flanker task (Table 3) were analyzed with a 2 (Design: Design 1 and Design 2) × 2 (VSTM: Low and High) × 2 (Distractor Compatibility: Compatible and Incompatible) ANOVA for repeated measures.

**Table 3:** Means of reaction times and error rates in the flanker task (Experiment 3)

Conditions	Distractor compatibility													
	Compatible					Incompatible					Interference			
	RT (ms)		Err (%)	Err (Arc)		RT (ms)	Err (%)		Err (Arc)		RT (ms)	Err (%)	Err (Arc)	
Low load (Design 1)	628.583 (122.987)		1.602 (2.182)		0.081 (0.100)	733.028 (146.554)		5.373 (3.900)		0.206 (0.115)	104.445 (70.228)	3.771 (4.108)		0.125 (0.139)
Low load (Design 2)	573.248 (138.212)		1.110 (1.556)		0.065 (0.085)	686.721 (167.905)		5.932 (6.223)		0.204 (0.144)	113.473 (75.286)	4.822 (6.255)		0.139 (0.155)
High load (Design 1)		629.529 (113.550)	2.087 (2.478)		0.101 (0.107)	747.937 (146.136)		7.154 (1.110)		0.246 (0.121)	118.407 (86.254)	5.067 (5.770)		0.145 (0.158)
High load (Design 2)		572.451 (159.403)	1.913 (3.242)		0.084 (0.114)	674.286 (172.539)		6.497 (6.493)		0.206 (0.163)	101.843 (78.893)	4.584 (6.322)		0.122 (0.169)

RT = reaction time, Err = error, Arc = arcsine transformation, ms = millisecond. Standard deviations of the means are given in parenthesis

According to the results, the main effect of the Design was significant (*F*(1, 27) = 14.812, *p* < 0.001, *ƞ2p* = 0.354). The RTs recorded under the Design 1 condition were greater than the RT recorded under the Design 2 condition (*MD* = 58.093; *SE* = 15.094). The main effect of the Distractor Compatibility was also significant (*F*(1, 27) = 63.129, *p* = 0.000, *ƞ2p* = 0.700), indicating that compared to the compatible condition, the reactions were slower under the incompatible conditions (*MD* = 109.540*, SE* = 12.361). The main effect of the VSTM load was non-significant (*F*(1, 27) = 0.005, *p* = 0.943, *ƞ2p* = 0.000).

Interaction effects were not significant (Design × VSTM load: *F*(1, 27) = 0.703, *p* = 0.409, *ƞ2p* = 0.025; Design × Distractor Compatibility: *F*(1, 27) = 0.126, *p* = 0.725, *ƞ2p* = 0.005, VSTM load × Distractor Compatibility: *F*(1, 27) = 0.018, *p* = 0.894, *ƞ2p* = 0.000; Design × VSTM load × Distractor Compatibility:* F*(1, 27) = 2.424*, p* = 0.131*, ƞ2p* = 0.082).

Four Bayesian analyses were conducted to investigate if the RT interference found in both low and high VSTM load conditions differed in each and between the two conditions of the “Design” variable. For all analyses, the alternative hypothesis was two-tailed and the prior parameter was Cauchy (*r* = 0.707).

- Design 1: Low VSTM load (*M* = 104.44, *SD* = 70.23) versus High VSTM load (*M* = 118.41, *SD* = 86.25) = BF01 = 2.52, %H = 0.023, *p* = *0.2*29, %95 GA = [-0.571, 0.145]
- Design 2: Low VSTM load (*M* = 113.47, *SD* = 75.29) versus High VSTM load (*M* = 101.84, *SD* = 78.89) = BF01 = 3.36, %H = 0.016, *p* = *0.3*61, %95 GA = [-0.194, 0.515]
- Low VSTM load: Design 1 versus Design 2 = BF01 = 3.84, %H = 0.012, *p* = *0.4*55, %95 GA = [-0.484, 0.222]
- High VSTM load: Design 1 versus Design 2 = BF01 = 2.82, %H = 0.021, *p* = *0.2*72, %95 GA = [-0.163, 0.551].

Accordingly, it was found that the available data provided anecdotal (BF01 = 0–3) and moderate level (BF01 = 3–10) evidence for the null hypothesis model (Jarosz & Wiley, 2014).

### Discussion

The third experiment, in which the option of using a verbal strategy in maintaining the visual representation was excluded, showed that VSTM load did not differentiate the distractors’ interference. This pattern was also valid for both levels of the Design variable. The amount of interference did not differ between the levels of the Design variable under any load condition. This finding even in a design where we were more confident that the memory task recruited VSTM resources, suggests that design-related differences between Experiment 1B of Konstantinou et al. (2014) and Experiment 3 of Yao et al. (2020) did not alter the results. Moreover, this experiment once again showed that the VSTM load did not modulate the distractor interference, consistent with our first two experiments.

We would like to emphasize a few points about the results of this experiment. First of all, it is noteworthy that the magnitude of the interference obtained in Experiment 3, which varied around 100 ms for each VSTM load condition, was higher than the magnitude of interference obtained in Experiments 1 and 2, which varied around 70 ms. It seems that the addition of the AS task increased the load on the cognitive control component, and thus may have increased the interference on the flanker task (Brand-D’Abrescia & Lavie, 2008; Lavie et al., 2004, Experiment 5).

There was a difference of approximately 50 ms in flanker RT between the levels of the Design variable. This difference was thought to be related to differences in experimental design. In the original experiment, the flanker stimulus display was presented for 150 ms, while the flanker response display was presented for 1,850 ms. In the experimental design of Yao et al. these durations were 200 and 1,800 ms, respectively (Yao et al., 2020; Experiment 3). In both tasks, response duration recording started with the arrival of the flanker response display. Thus, in Yao et al.'s experimental design, the recording of participants' responses started with a delay of 50 ms, resulting in RTs appearing 50 ms faster on average. This difference in average RT was not thought to be critical for the compatibility effect, since it was present in both compatible and incompatible trials. Indeed, Yao et al., (2020, Experiment 7) varied the presentation time of the flanker task and observed no difference in the level of the compatibility effect despite differences in RTs. The fact that this difference in flanker RT in Experiment 3 was not observed in the analysis comparing data from Experiments 1 and 2 showed the importance of using a within-subject approach in such experiments with dependent variables changing in milliseconds.

Another issue was the unexpected loss of data (50%) in the third experiment. While no participant was eliminated in Experiment 1 due to exceeding the 50% error rate in the memory task, only one participant was eliminated in Experiment 2 because of this. In terms of the eliminations related to Cowan's *K* values, on the other hand, five participants were eliminated in both Experiment 1 and Experiment 2. Similarly, in Experiment 3, no one was eliminated in the Design 1 condition for making more than 50% errors in the memory task, while two people were eliminated in the Design 2 condition. However, in Experiment 3, we encountered a different picture in terms of other elimination criteria.

There were five participants whose high and low load Cowan's *K* value difference was not above "zero" in the Design 1 condition, while 16 participants were in the same situation in the Design 2 condition. As previously reported, the number of participants eliminated because of this criterion in the first two experiments was five for each experiment.

When the number of data lost at the levels of the Design variable in Experiment 3 was compared with the number of data lost in Experiments 1 and 2, it was noticeable that the main difference emerged in the Design 2 condition. In other words, the AS manipulation introduced in Experiment 3 might have negatively affected working memory performance more under the Design 2 conditions, especially when the VSTM load was high. This could mean that verbal memory support may have been already less in Experiment 1 than in Experiment 2. Perhaps the fact that the colored squares were presented smaller and for shorter durations, which may have been perceptually more demanding, may have caused verbalization to take a back seat in Experiment 1. On the other hand, in Experiment 2, where the colors were both larger and presented for a longer duration, the likelihood of utilizing verbalization may have increased. In Experiment 3, the AS task may have impaired the performance of the participants under the high load/Design 2 condition. Therefore, it is possible that the difference in the rate at which VSTM memory capacity is occupied in the high load condition may have had some effect on the results obtained in Experiment 3. However, given that the participants were exposed to all experimental conditions and there was no difference between the two VSTM load conditions at any level of the “Design” variable, the small difference between the two levels of the Design variable in terms of VSTM capacity occupancy in the high VSTM load condition was not considered to have a confounding effect on the results.

Considering that the loss of a large number of participants may have an impact on the results, we re-analyzed the data of Experiment 3 with lax criteria, just as we reanalyzed Experiment 1. We have included detailed information on the results in the OSM under the heading of “*Lax Criteria Analyses of Experiment 3.”* These additional analyses indicated that the VSTM load did not have a modulatory effect on distractor interference in terms of both RT and error rates. This pattern was also observed for both experimental designs. In other words, even when the elimination criteria were kept flexible, the load effect was insignificant on distractor interference and the pattern of this effect did not change according to the design.

The results of the third experiment also showed that the explanation for why we were unable to replicate the critical finding of the original study (Experiment 1B of Konstantinou et al., 2014) may not be linked to the freedom in the use of verbal or visual strategies.

To carry out another test, we used a data-driven method to identify if there was a variable that may have had an impact on the differing results. In this approach, we used the data of Experiment 1 but separated the participants who showed a perceptual load effect pattern (participants whose level of interference effect in the high VSTM load condition was lower than their level of interference effect in the low VSTM load condition) from those who did not show such pattern. To strengthen the distinction between the participant groups, we selected the participants in the first and last quartiles when ranked from small to large according to the difference (high – low load) in interference effect between load conditions. We examined which dependent measures differed as a function of group (Load Pattern Group: Perceptual and Other) by analyzing the data of the 32 participants (the 16 participants who showed the strongest perceptual load effect and the 16 participants who did not show the perceptual load effect). The results are also provided in the OSM in the section “*VSTM Load Pattern Group Analyses.”* As a result of these analyses, it was revealed that the participants who showed the perceptual load effect pattern had higher Cowan's *K* values in the high load condition than the other participants. In other words, their capacities were filled more by loading VSTM. Moreover, although the effect was not statistically significant, the participants showing a perceptual load effect pattern reacted faster for the target in the flanker task than the other group of participants in all conditions. Considering that both giving faster reactions and having more capacity load might have increased perceptual demand, the combination of these two factors may be effective in observing the perceptual load effect.

We examined the relationship of these two factors with the interference effect for the first two experiments with correlation analyses. For just Experiment 1, we reported a significant negative correlation between the amount of change in the Cowan's *K* value and the difference in the interference effect between the two load conditions (see *“Correlations”* in the OSM). All these findings are discussed in the *General discussion*.

## General discussion

The main purpose of this study was to test if certain differences in the task design of the replication attempt (Experiment 3 of Yao et al., 2020), which reported different results from the original study (Experiment 1B of Konstantinou et al., 2014), could be responsible for the inconsistent findings. In line with this main purpose, we conducted exact replications of these two experiments; the results of the analyses of variance in both our experiments showed that the VSTM load had no effect on reducing the undesirable effects of the distractors. The results are in line with the findings of Yao et al. (2020), which demonstrated with nine different experiments that VSTM load did not modulate distractor interference. The fact that our experimental results were consistent with each other and the findings of Yao et al. (2020) but inconsistent with the results of Konstantinou et al. (2014) prompted us to carry out additional analyses to investigate possible reasons for the inconsistency between our Experiment 1 and Experiment 1B of Konstantinou et al. (2014).

In order to make the results more clearly attributable to the design differences, we used strict and identical elimination criteria in all experiments, which differed from the criteria in the original studies. For this reason, we investigated the effect of the strictness of the criteria on the results of Experiment 1 by re-analyzing the data using flexible criteria. We found that the pattern of results remained unchanged.

We also reasoned that the participants’ strategic freedom in maintaining memory items can possibly explain why we did not obtain a perceptual load pattern in our Experiment 1 and why the design differences were ineffective. Christophel et al. (2017) emphasized a distributed nature of WM and suggested that one of the most important factors determining the storage areas of representations on the cortex is the level of verbalization and abstractness. Any representation is stored in the frontal areas when it is coded verbally, categorically, or abstractly. Visual, or concrete detailed representations, on the other hand, are stored in visual/sensory areas (Lee et al., 2013; Yan et al., 2021). Considering this cortical difference, we designed a third experiment in which we prevented the maintenance of memory content by verbal codes through articulatory suppression, and re-tested the effects of design differences. The results again showed that the VSTM load did not alter the distractor interference effect. This was the same for each experimental design, showing that freedom of strategy did not change the results.

For the inconsistency between the results of Experiment 1 and the original experiment (Experiment 1B of Konstantinou et al., 2014), we also conducted a new analysis by separating the participants who showed the perceptual load effect most strongly from those who did not in Experiment 1’s data. As a result of this examination with the most representative 32 participants’ data, we found that the Cowan's *K* values of the participants who showed the perceptual load effect pattern were higher than those of the other participants in the high VSTM load condition. Moreover, although it was not significant, the participants showing perceptual load pattern reacted faster to the flanker target than the other group in all conditions.

In line with these findings, we conducted correlation analyses for our first two experiments, predicting that there would be a negative correlation between the amount of memory capacity filled and the difference in the interference effect between the two load conditions (see *“Correlations”* in the OSM). In Experiment 2, the interference difference obtained by subtracting the interference effect at high load from the effect at low load was not significantly correlated with the Cowan's *K* difference (*r* = -0.015, *p* = 0.454, one-tailed). On the other hand, the same correlation analysis for Experiment 1 yielded a significant result (*r* = -0.244, *p* = 0.026, one-tailed), and it was observed that the difference in the amount of interference effect between the two load conditions decreased with increasing capacity load.

This correlation analysis result, which was consistent with the perceptual load effect revealed by Konstantinou et al. (2014), prevented us from concluding definitively that the VSTM load did not make a difference on the distractor interference effect. The absence of such a relationship in Experiment 2, on the other hand, led us to suspect that design-related differences might have affected the correlation analyses. The shorter retention interval, the smaller colored squares, and the shorter flanker display may have enabled the perceptual demand of the original study (Experiment 1B of Konstantinou et al., 2014) to be at a higher level, which in turn may have been reflected in the correlation analysis. However, this effect did not appear in the factorial variance analyses. Considering the results of the correlation analysis, we suggested that the loading was not strong enough to show up in variance analysis.

Although we argued that Experiment 1B of Konstantinou et al. (2014) had a number of design features that facilitated the acquisition of the perceptual load pattern, it remains to be explained why the significant interaction effect in the original study was not obtained in our Experiment 1. The findings of our experiments and additional analyses revealed that the differences in results were not related to the differences in verbalization possibility or elimination criteria. On the other hand, our additional findings showing the importance of *the amount of capacity filling*, *the speed of the response*, and *the perceptual characteristics of the task* led us to suggest that there may have been differences between the original study and our experiment in these respects.

Given that the instructions for the flanker task emphasize reacting as fast as possible, response speed can be considered as an indicator of success in adhering to the task instruction. There might have also been a difference in participants’ adherence to task instruction between our replication attempt and the original study. Although there is no difference between the two studies in terms of average RTs, considering that different participants may have different baseline reaction speeds, it is still possible that participants in the original study may have been more adherent to the task instructions. However, considering the existence of previous studies that did not show a modulation effect of WM load, it does not seem very reasonable to think that the difference in the rate of compliance with the instruction was a strong reason for explaining the inconsistency.

Another possibility was that the participants in the original study may have had lower perceptual or visual working memory capacity than the participants in our study. Thus, it may have been easier to observe the perceptual load effect when loading the limited capacity in the original study.

In summary, the following can be said about the modulation effect of the VSTM load on distractor interference. The resources for maintaining visual working memory content and the resources associated with visual perception may be the same, as suggested by the sensory recruitment model for which there is a lot of support (Christophel et al., 2018; Harrison & Tong, 2009; Lee et al., 2013; Lorenc et al., 2020; Riggall & Postle, 2012). From this point of view, a high VSTM load is expected to consume the resources of the sensory areas, which would be used for the processing of the intervening flanker task. However, in our experiments we consistently found that loading the capacity with one and four stimuli did not cause a sufficient difference.

According to results of all our experiments and the additional analyses, we speculated that at least two factors seem to be crucial for increasing the possibility of visual working memory to produce a perceptual load-like effect. *The first of these is sufficiently strong filling of the visual working memory load capacity* (a sufficiently large difference in loading between the two conditions). The absence of significant correlations in the second experiment also led us to infer that not only working memory loading but also *perceptual demands (load) of the whole task* (short retention interval, small stimuli, etc.) may have contributed to the outcome effect. Therefore, another factor important in producing a perceptual load-like effect might be higher perceptual demand of the whole task. In short, it might be that in order to see the perceptual load-like effect, the shared capacity of the visual perception/visual short-term memory must be filled as much as possible, perceptually and/or by strong loading of visual working memory. Otherwise, the remaining perceptual capacity may still be high enough to process distractors in both low and high load conditions, and this might be the reason for our non-significant interactions found in all three experiments.

To conclude, the design differences between these two experiments with inconsistent results did not seem to be strong enough to explain the differences in the results obtained in the analysis of variances. However, it was seen that the design differences somehow contribute to the reduction of the interference effect by changing the perceptual demands of the tasks. It is worth noting that the variables, which our experiments and additional analyses revealed to be critical for the perceptual load effect, were not directly manipulated in this study. Such an investigation in future studies will be enlightening for understanding the interaction between VSTM load, perceptual load, and selective attention.

## Supplementary Information

Below is the link to the electronic supplementary material.

Supplementary file1 (DOCX 1008 kb)

## Data Availability

The raw data of all three experiments are available via the Open Science Framework at: https://osf.io/3gndt/files/osfstorage

## Footnotes

1 We corresponded with Dr. Konstantinou, and were informed that only three of 22 participants in Experiment 1B of his study (Konstantinou et al., 2014) were over the age of 30 years (35, 43, and 51 years, respectively). For this reason, the age range for this planned replication study was determined as 18–30 years. This age range was determined as a criterion in all experiments carried out within the scope of this study.

2 The flanker response keys, which were defined as “0” and “2” on the Numpad in the original study, were changed to prevent data loss since E-Prime stops the Numpad activation from time to time. For this reason, the flanker response keys were defined as “1” and “2” among the numbers arranged from left to right at the top of the keyboard. We corresponded with Dr. Konstantinou on that issue, and learned that the critical factor in determining the response keys was only to position both hands far enough from each other to ensure comfortable responses. Accordingly, the memory task response keys, which were defined as “A” and “S” in the original study, were determined as “8” and “9” from the numbers on the upper part of the keyboard. Thus, both hands were positioned as far from each other as in the original work. The design of these response keys was the same for all experiments we conducted throughout our study.

3 Dr. Konstantinou informed us that each trial started with the participant response, which reduced fatigue and the number of errors.

4 It has not been clarified what the color space of the original work was. When the original color codes were examined, it was thought that the color space could be the Yxy. However, two of the original color codes (reported for white and background gray) did not produce the desired white and gray color in Yxy. Therefore, white color and gray color were determined in RGB space. The equivalents of these newly determined white (RGB: 255, 255, 255) and gray (RGB: 180, 180, 180) colors in Yxy space were x = .31, y = .33, 100 cd/m^2^ and x = .31, y = .33; 45.64 cd/m^2^, respectively. It should be emphasized that in Yao et al.'s (2020) replication attempt, all of the color codes were determined in RGB space, unlike Experiment 1B of Konstantinou et al. (2014).
