# Supplementary material for: Load effect of visual working memory on distractor interference: An investigation with two replication experiments
Source: Mem Cognit. 2024 Jul 22;53(3):832–52. doi: 10.3758/s13421-024-01610-y (PMC12053181; doi:10.3758/s13421-024-01610-y)
Supplement: Supplementary file 1 — Supplementary file1 (DOCX 1008 kb) [file 13421_2024_1610_MOESM1_ESM.docx]

**Supplementary Material**

**Load effect of Visual Working Memory on Distractor Interference. An Investigation with Two Replication Experiments.**

1. ***Lax Criteria Analyses of Experiment 1***

We conducted new analyses, considering that the reason why the results of Konstantinou et al. (2014) could not be reproduced might have been related to the strict elimination criteria. In these new analyses, we kept the criteria loose. That is, we did not apply elimination criteria based on Cowan's K and the number of trials. The results of these additional analyses, including data from 64 participants, were provided below.

The result of the paired samples *t*-test showed a significant difference between two VSTM load conditions in terms of the Cowan’s K values (Low VSTM Load: *M* = 0.909, *SD* = 0.073; High VSTM Load: *M* = 1.980, *SD* = 0.765; *p* < .001, *Cohen’s D* = 1.971)

Arcsine transformed error rates of the flanker task was analyzed by 2 (VSTM Load: Low and High) x 2 (Distractor Compatibility: Compatible and Incompatible) ANOVA for repeated measures. Results indicated neither main effect of the VSTM load (*F*_(1, 63)_ = 1.746, *p* = .191, *n^2^_p_* = .027) nor the two-way interaction (*F*_(1, 63)_ = 1.700, *p* = .197, *n^2^_p_* = .026) was significant. As expected, the main effect of distractor compatibility, on the other hand, was significant (*F*_(1, 63)_ = 88.351, *p* < .001, *n^2^_p_* = .584) which indicated that the error rates were higher for incompatible trials (*MD* = 0.111, *SE* = 0.012).

Reaction times of the flanker task were also analyzed by 2 (VSTM Load: Low and High) x 2 (Distractor Compatibility: Compatible and Incompatible) ANOVA for repeated measures. Results were similar to the results of the analyses on error rates and revealed that the main effect of VSTM load was not significant (*F*_(1, 63)_ = 0.003, *p* = .955, *n^2^_p_* = .000). The main effect of distractor compatibility, on the other hand, was significant (*F*_(1, 63)_ = 201.011, *p* < .001, *n^2^_p_* = .761) indicating that the reactions were faster for compatible trials (*MD* = 72.909, *SE* = 0.012). Importantly, two-way interaction was not significant (*F*_(1, 63)_ = 2.237, *p* = .140, *n^2^_p_* = .034, Figure S1) showing that there was no difference between the low VSTM load condition (*M =* 69.316, *SD =* 41.946) and under the high VSTM load condition (*M =* 76.502, *SD =* 48.625) in terms of the amount of the interference.

***Figure S1***

1. ***Lax Criteria Analyses of Experiment 3***

The data of two participants whose error rate was above the chance level in any task were removed from the dataset and the analyses were conducted using the data of 54 out of 56 participants.

According to the results of 2 (Design: Design 1 and Design 2) x 2 (VSTM Load: Low and High) ANOVA for repeated measures on Cowan’s K values, all the effects were statistically significant.

The main effect of Design (*F*_(1, 53)_ = 30.363, *p* < .001, *n^2^_p_* = .364) showed that the mean Cowan’s K value was lower under the Design 2 condition than the value under the Design 1 condition (*MD* = 0.212, *SE* = 0.038). The main effect of VSTM Load (*F*_(1, 53)_ = 59.817, *p* < .001, *n^2^_p_* = .530) revealed that the mean Cowan’s K value was lower under the low VSTM load condition compared to the other load condition as expected (*MD* = 0. 643, *SE* = 0.083).

Moreover, Bonferroni corrected pairwise comparisons for the significant interaction effect (*F*_(1, 53)_ = 32.784, *p* < .001, *n^2^_p_* = .382) indicated that the difference between two load conditions were significant for each level of the Design variable (Design 1: *MD* = 0. 867, *SE* = 0.092, *p* < .001; Design 2: *MD* = 0. 419, *SE* = 0.092, *p* < .001). It was also revealed that compared with the value under the Design 2 condition, the mean Cowan’s K value was higher under the Design 1 condition when the VSTM load was high (*MD* = 0. 436, *SE* = 0.055, *p* < .001) but not when the load was low (*MD* = 0. 012, *SE* = 0.055, *p* = 1.000). As in the original analyses reported in the article, it was drawn attention that compared to Design 2, the capacity was loaded more in Design 1 under the high VSTM load condition.

We run 2 (Design: Design 1 and Design 2) x 2 (VSTM Load: Low and High) x 2 (Distractor Compatibility: Compatible and Incompatible) ANOVA for repeated measures on the arcsine transformed **error rates** in flanker task.

Results indicated that the main effect of the VSTM Load (*F*_(1, 53)_ = 9.626, *p* = .003, *n^2^_p_* = .154) was significant and showed that mean error rate was higher under the high load condition (*MD* = 0.027, *SE* = 0.009). It was also found that the main effect of Distractor Compatibility was also significant (*F*_(1, 53)_ = 102.913, *p* < .001, *n^2^_p_* = .660). The error rate was higher under incompatible condition as expected (*MD* = 0.162, *SE* = 0.016).

All the other effects were not significant [Main effect of Design: (*F*_(1, 53)_ = 0.274, *p* = .603, *n^2^_p_* = .005); Interaction of Design x VSTM Load: (*F*_(1, 53)_ = 1.914, *p* = 0.172, *n^2^_p_* = .035); Interaction of Design x Distractor Compatibility: (*F*_(1, 53)_ = 0.455, *p* = .503, *n^2^_p_* = .009); Interaction of VSTM Load x Distractor Compatibility (*F*_(1, 53)_ = 0.909, *p* = .345, *n^2^_p_* = .017); Three-way interaction: (*F*_(1, 53)_ = 0.532, *p* = .469, *n^2^_p_* = .010)].

We also run 2 (Design: Design 1 and Design 2) x 2 (VSTM Load: Low and High) x 2 (Distractor Compatibility: Compatible and Incompatible) ANOVA for repeated measures on the **reaction times** of the flanker task.

Results indicated that the main effect of the Design (*F*_(1, 53)_ = 22.124, *p* < .001, *n^2^_p_* = .294) was significant indicating that mean reaction time was higher under the Design 1 condition (*MD* = 50.949, *SE* = 10.832). We already discussed that this difference was because of the difference in durations of flanker response display between two task designs. It was also found that the main effect of Distractor Compatibility was also significant (*F*_(1, 53)_ = 92.764, *p* < .001, *n^2^_p_* = .636). The reactions were faster under compatible condition as expected (*MD* = 131.913, *SE* = 13.696).

All the other effects were not significant [Main effect of VSTM Load: (*F*_(1, 53)_ = 0.033, *p* = .856, *n^2^_p_* = .000); Interaction of Design x VSTM Load: (*F*_(1, 53)_ = 2.899, *p* = 0.094, *n^2^_p_* = .052); Interaction of Design x Distractor Compatibility: (*F*_(1, 53)_ = 0.540, *p* = .466, *n^2^_p_* = .010); Interaction of VSTM Load x Distractor Compatibility (*F*_(1, 53)_ = 0.218, *p* = .643, *n^2^_p_* = .004); Three-way interaction: (*F*_(1, 53)_ = 0.396, *p* = .532, *n^2^_p_* = .007, Figure S2)].

***Figure S2***

These findings showed that having lax or strict elimination criteria did not change the pattern of the results. In addition, it was once again shown that the VSTM load did not modulate the distractor interference for both experimental designs.

1. ***VSTM Load Pattern Group Analyses***

For these analyses, a difference value was first obtained by subtracting the interference effect in RT in the low load condition from the interference effect in the high load condition. Participants with a negative difference value were coded as participants showing a perceptual load pattern, while the others were coded as others. Accordingly, 30 of the 64 participants in the data set showed the perceptual load pattern, while 34 did not. We thought that comparing the participants who have strong perceptual load pattern, and those who did not have would be more effective in showing the possible differences between the two groups. For this reason, the data of 32 people were included in the analyses by coding the 16 people in the first quartile with the lowest difference score when ranked according to the interference effect difference as people with a strong perceptual load pattern and the 16 people in the last quartile as others.

First of all, to show that these two groups really differ in terms of the pattern of the interference effect, we conducted a 2 (VSTM Load: Low and High) x 2 (Load Pattern Group: Perceptual and Other) mixed ANOVA analysis on the amount of interference effect.

This analysis revealed significant main effect of the VSTM Load (*F*_(1, 30)_ = 18.236, *p* < .001, *n^2^_p_* = .378) which indicated that the interference effect was higher in the high VSTM load condition (*MD* = 10.523, *SE* = 2.464). The main effect of Load Pattern Group, on the other hand, was not significant (*F*_(1, 30)_ = 2.760, *p* = .107, *n^2^_p_* = .084). Importantly, and as expected, the interaction effect was significant (*F*_(1, 30)_ = 407.902, *p* < .001, *n^2^_p_* = .931).

According to the Bonferroni corrected pairwise comparisons while the difference between the two groups under the low VSTM load condition was not significant (*MD* = 24.273, *SE* = 15.543, *p* = .770), it was significant under the high VSTM load condition (*MD* = 75.261, *SE* = 15.543, *p* < .001). Moreover, the difference between the two VSTM load condition was significant for both the group showing perceptual load pattern (*MD* = 39.244, *SE* = 3.485, *p* < .001), and the other group (*MD* = 60.290, *SE* = 3.485, *p* < .001, Figure S3).

Cowan's K values were analyzed with 2 (VSTM Load: Low and High) x 2 (Load Pattern Group: Perceptual and Other) mixed ANOVA.

***Figure S3***

According to the results of the analysis on Cowan’s K, the main effect of VSTM load (*F*_(1, 30)_ = 116.634, *p* < .001, *n^2^_p_* = .795) was significant as expected. Accordingly, the Cowan’s K value under the high load condition was higher than the score under the low load condition (*MD* = 1.194, *SE* = 0.111). Interestingly, the main effect of the load pattern group was also found to be significant (*F*_(1, 30)_ = 8.214, *p* = .008, *n^2^_p_* = .215) and this effect revealed that the mean Cowan’s K value of the participants in the perceptual load group was higher than the mean value of the other group (*MD* = 0.342, *SE* = 0.119). Moreover, interaction effect was also significant (*F*_(1, 30)_ = 6.561, *p* = .016, *n^2^_p_* = .179, Figure S4).

Bonferroni corrected pairwise comparisons indicated that not in the low (*MD* = 0.059, *SE* = 0.163, *p* = 1.000) but in the high load condition the mean Cowan’s K values differed significantly (*MD* = 0.625, *SE* = 0.163, *p* = 0.002) as a function of load pattern group. This suggests that one factor that may determine the load effect pattern may be the amount/level of loading. It can be argued that the perceptual load effect is likely to emerge more clearly as the difference in occupied capacity increases between the two load conditions.

***Figure S4***

*Note*. Error bars show standard errors

Arcsine transformed error rates of the flanker task was analyzed by 2 (VSTM Load: Low and High) x 2 (Distractor Compatibility: Compatible and Incompatible) x 2 (Load Pattern Group: Perceptual and Others) mixed ANOVA. Results indicated that error rates were higher under the incompatible conditions than the rate under the compatible condition (*MD* = 0.129, *SE* = 0.018) and this difference was significant (*F*_(1, 30)_ = 53.932, *p* < .001, *n^2^_p_* = .643). All other effects were not statistically significant.

Reaction times of the flanker task was also analyzed by 2 (VSTM Load: Low and High) x 2 (Distractor Compatibility: Compatible and Incompatible) x 2 (Load Pattern Group: Perceptual and Others) mixed ANOVA. The main effect of distractor compatibility was significant (*F*_(1, 30)_ = 95.220, *p* < .001, *n^2^_p_* = .760) which indicated that the reactions were faster for compatible trials (*MD* = 74.876, *SE* = 7.673). Although, mean reaction times for the perceptual load group were 44 ms smaller than the mean reaction times for the other group, this difference was not significant (*F*_(1, 30)_ = 1.084, *p* = .306, *n^2^_p_* = .035).

The interaction of VSTM load and the distractor compatibility was significant (*F*_(1, 30)_ = 18.237, *p* < .001, *n^2^_p_* = .378) and Bonferroni corrected pairwise comparisons indicated that in both load conditions reactions to incompatible trials were slower (low load: *MD* = 69.614, *SE* = 7.772, *p* < .001; high load: *MD* = 80.137, *SE* = 7.772, *p* < .001). However, there was no difference between two load conditions under both compatible and incompatible conditions (*p* = 1.000 for each condition). The three-way interaction was also found to be significant (*F*_(1, 30)_ = 407.908, *p* < .001, *n^2^_p_* = .931). Although there was no significant difference between the groups in terms of reaction time in low-load compatible (*MD* = 41.805, *SE* = 44.076, *p* = 1.000), high-load compatible (*MD* = 20.976, *SE* = 44.076, *p* = 1.000), low-load incompatible (*MD* = 17.532, *SE* = 44.076, *p* = 1.000), and high-load incompatible conditions (*MD* = 96.237, *SE* = 44.076, *p* = 1.000), it is noteworthy that in all comparisons, the group showing perceptual load effect responded faster than the other group and this difference was high up to 96 ms in the high-load incompatible condition.

The fact that this speed difference was in same direction in all conditions may be an indication that the participants who showed a perceptual load effect adhered more to the flanker task instruction. Therefore, another factor determining the modulation of the VSTM load on the distractor interference effect may be the reaction time, which may be related to the level of participants' adherence to the instruction.

As a result of these analyses, it was revealed that the participants who showed the perceptual load effect pattern had higher Cowan's K scores than the other group of participants in the high load condition. In other words, their VSTM capacities were filled more by loading VSTM. It was also revealed that although the effect was not statistically significant, the participants showing perceptual load effect pattern reacted faster to the flanker target than the other group of participants in all conditions. The combination of these two factors may be crucial in whether or not the VSTM load have a modulation effect on distractor interference.

1. *Correlations*

We investigated the relationship between the level of interference, the reaction speed and the level of capacity loading, which were identified as potentially important factors in “VSTM Load Pattern Group Analyses”. We set our dependent variable as a difference score obtained by subtracting the interference effect in the high VSTM load condition from the effect in the low VSTM load condition. The level of VSTM capacity loading was also a difference score calculated by subtracting the Cowan’s K value in the high load conditions from the value in the low load condition. The reaction speed variable was the mean reaction time in flanker task. We examined the correlations between these variables.

We formed two one-way hypotheses. Accordingly, we expected that as the average reaction time increases, the difference in interference effect will also increase. Our other hypothesis was that as the Cowan's K difference (VSTM capacity loading) increases, the interference deference will decrease. We performed these correlation analyses for both Experiment 1 (N = 64) and Experiment 2 (N = 60), which had different design features.

For the Experiment 2, the correlation between the interference difference and both average reaction time (*r* = .094, *p* = .238, one-tailed) and the Cowan’s K difference (*r* = -.015, *p* = .454, one-tailed) was not significant. For the Experiment 1, the correlation between average reaction time and interference difference was also not significant (*r* = .164, *p* = .098, one-tailed, Figure S5)

***Figure S5***

On the other hand, the correlation between the Cowan’s K difference and interference difference was significant (*r* = -.244, *p* = .026, one-tailed, Figure S5). According to this significant correlation, as the level of VSTM capacity occupation increases, the difference in distractor interference between the two load conditions decreases. However, this pattern was not reflected in the ANOVA results reported in *"Lax Criteria Analyses for Experiment 1*" which was also conducted with same data. According to correlation analysis, the perceptual load pattern (*the difference in interference (High – Low Load) falls below zero*) seems to be obtained when the capacity being sufficiently filled. Otherwise, the residual perceptual capacity seems to be sufficient to cope with the distractor effect at a similar level in both VSTM load conditions as can be seen in our three experiments.

*Note*. Error bars show standard errors

*Note*. Error bars show standard errors
